# Open Notes in Swedish Psychiatric Care (Part 2): Survey Among Psychiatric Care Professionals

**DOI:** 10.2196/10521

**Published:** 2018-06-21

**Authors:** Lena Petersson, Gudbjörg Erlingsdóttir

**Affiliations:** ^1^ Department of Design Sciences Faculty of Engineering Lund University Lund Sweden

**Keywords:** electronic health records, eHealth, telemedicine, postimplementation survey, health care surveys, mental health, Open Notes, psychiatry, health professionals

## Abstract

**Background:**

This is the second of two papers presenting the results from a study of the implementation of patient online access to their electronic health records (here referred to as Open Notes) in adult psychiatric care in Sweden. The study contributes an important understanding of both the expectations and concerns that existed among health care professionals before the introduction of the Open Notes Service in psychiatry and the perceived impact of the technology on their own work and patient behavior after the implementation. The results from the previously published baseline survey showed that psychiatric health care professionals generally thought that Open Notes would influence both the patients and their own practice negatively.

**Objective:**

The objective of this study was to describe and discuss how health care professionals in adult psychiatric care in Region Skåne in southern Sweden experienced the influence of Open Notes on their patients and their own practice, and to compare the results with those of the baseline study.

**Methods:**

We distributed a full population Web-based questionnaire to psychiatric care professionals in Region Skåne in the spring of 2017, which was one and a half years after the implementation of the service. The response rate was 27.73% (699/2521). Analyses showed that the respondents were representative of the staff as a whole. A statistical analysis examined the relationships between health professional groups and attitudes to the Open Notes Service.

**Results:**

A total of 41.5% (285/687) of the health care professionals reported that none of their patients stated that they had read their Open Notes. Few health care professionals agreed with the statements about the potential benefits for patients from Open Notes. Slightly more of the health care professionals agreed with the statements about the potential risks. In addition, the results indicate that there was little impact on practice in terms of longer appointments or health care professionals having to address patients’ questions outside of appointments. However, the results also indicate that changes had taken place in clinical documentation. Psychologists (39/63, 62%) and doctors (36/94, 38%) in particular stated that they were less candid in their documentation after the implementation of Open Notes. Nearly 40% of the health care professionals (239/650, 36.8%) reported that the Open Notes Service in psychiatry was a good idea.

**Conclusions:**

Most health care professionals who responded to the postimplementation survey did not experience that patients in adult psychiatric care had become more involved in their care after the implementation of Open Notes. The results also indicate that the clinical documentation had changed after the implementation of Open Notes. Finally, the results indicate that it is important to prepare health care professionals before an implementation of Open Notes, especially in medical areas where the service is considered sensitive.

## Introduction

### Background

This papers is the second of 2 that present the results from a study of the pre- and postimplementation of patient online access to their electronic health records (here referred to as Open Notes) in adult psychiatric care in Sweden. The study consisted of 2 surveys: 1 at baseline and 1 at postimplementation. In this paper, we present the results from the postimplementation survey and compare them with the results from the previously published baseline survey [1]. The study, as a whole, is unique and contributes an important understanding of the expectations and concerns that existed among Swedish health care professionals (HCPs) before the introduction of Open Notes in psychiatry and their perceptions of the impact of the technology on their own work and on patient behavior afterward. The study design also allowed us to compare expectations and experiences between different groups of HCPs.

### Open Notes in Sweden

The Open Notes Service was first launched for patients in nonpsychiatric care in Uppsala County in 2012. Today, all citizens in the country except adolescences between 13 and 16 years of age can read their Open Notes from nonpsychiatric settings online. In some counties, however, patients in psychiatric care can also read their notes online. Region Skåne, the site of our study, was the first county to add psychiatric care to the service [1]. Open Notes is an important civic eHealth service in Sweden and, as in other countries, the intention is to increase patient empowerment and participation [2]. Health care coverage in Sweden is universal, which means that all residents have access to publicly financed health care and, thus, the Open Notes Service. Sweden has 10 million citizens and, at the end of April 2018, approximately 1.8 million of them had read their Open Notes online. Swedish patients logged in to the service nearly 5.4 million times during the first 4 months of 2018, which is an average of almost 49,000 log-ins every day. Every day during these 4 months, 2500 patients used the service for the first time. However, the service is not seen as entirely positive among HCPs; results from qualitative studies in Sweden indicated that some doctors have a negative view of the service [3,4].

In part 1 of this study, carried out in 2015, we queried HCPs in Region Skåne in a baseline survey about how they expected Open Notes to affect their patients and their own practice before its implementation in adult psychiatric care [1]. We later followed up with a postimplementation survey to gain more knowledge about how the HCPs in adult psychiatric care in Region Skåne experienced the influence that Open Notes had on their patients and their own practice.

### Principal Findings From the Baseline Survey

The results from the full population baseline survey showed that psychiatric HCPs generally thought that Open Notes would influence both the patients and their own practice negatively. Doctors, psychologists, and medical secretaries were in many cases more negative than nurses and assistant nurses. Almost 60% of the HCPs believed that patients who read their Open Notes would be more worried, and half of the respondents thought that these patients would find the notes more confusing than helpful. To our surprise, medical secretaries were as negative toward the service as the doctors were. In particular, their concerns were that patients would be offended by the entries and would disagree with the content in their records. However, the respondents also believed that Open Notes would benefit the patients. Approximately 40% anticipated that most of the patients would feel more in control of their care, and 30% believed that patients would be better prepared for appointments. One of the most notable results in the baseline study was that approximately 60% of both doctors and psychologists were worried that they would be less candid in their documentation after the implementation of Open Notes [1].

We sent out a second survey to investigate how the concerns listed above and other opinions of the HCPs had changed after the implementation of patient access to their Open Notes in psychiatry. We queried the HCPs about their experiences of the service one and a half years after the baseline survey and, thus, one and a half years after the implementation. The objective of this study was to describe and discuss how HCPs in adult psychiatric care in Region Skåne in southern Sweden experienced the influence that the Open Notes Service had on their patients and their own practice, and to compare the results with those from the previously published baseline survey study.

## Methods

### Survey Design

The material we present here is the result of a postimplementation survey in psychiatric care. Both this survey and the previous baseline survey were part of a larger research project (the eHealth Services’ Impact on the Working Environment of Health Professionals [EPSA] Project, financed by AFA Insurance, Sweden) on how the work and work environment of HCPs are influenced by civic eHealth services such as Open Notes. The employees in adult psychiatry were not presented the results from the baseline survey until after they had completed the postimplementation survey in order to avoid biasing the respondents’ opinions. We distributed the postimplementation survey one and a half years after the implementation of Open Notes. The reason for this decision was that we wanted both the staff and patients to have gained considerable experience from using the service before we sent the follow-up survey.

Both the baseline [1] and the postimplementation surveys were based on the surveys developed by the OpenNotes Project in the United States [5-8]. In both cases, the original surveys were translated and adjusted to fit the Swedish context. The questions were then translated into English for presentation in this paper. The postimplementation survey covers the following themes: benefits and risks for patients, changes in practice, changes in clinical documentation and work conditions, about me, and future development of Open Notes. Most of the questions from the baseline survey remained the same, but we changed the verb tense in many of them. We also changed the response options in some questions either because some professional groups would otherwise not have been able to answer the specific question, or because we were interested in a more detailed answer than the response options from the baseline survey offered. In addition, because Open Notes in adult psychiatric care in Region Skåne has been implemented in the universal health care system, patients with many different diagnoses can read their records. A question posed in the discussion of the first paper was whether there was a difference in how the service influenced patient groups with various diagnoses. Did HCPs think that Open Notes could benefit any special group of patients or might the service be particularly problematic for other groups of patients in psychiatry [1]? We thus added this question to the postimplementation survey. The survey consisted of 44 fixed-choice questions and 20 open-ended questions. We designed the postimplementation survey so that the respondents could choose not to answer all the questions.

### Setting and Population

The Division of Psychiatric Care in Region Skåne consists of 3 subdivisions: adult, child and youth, and forensic. It employs roughly 3000 people. In 2017, there were over 575,000 appointments, of which almost one-fifth were with a doctor. The number of unique patients was over 56,000. Patients in adult psychiatry were offered online access to their Open Notes in October 2015, and the plan was that patients in forensic psychiatry and parents of patients in child and youth psychiatry should be offered the service from the fall of 2018.

We invited the entire population of HCPs (n=2521) in adult psychiatry in Region Skåne who meet patients to participate in this postimplementation study. This included assistant nurses, doctors, medical secretaries, nurses, occupational therapists, physical therapists, psychologists, social workers, and unit managers. In the population, approximately two-thirds of the doctors were psychiatrists, and nearly half of the nurses were specialists in psychiatric care. We referred to these two professions as doctors and nurses in both questionnaires. The rationale for not taking a sample was that the employees are a heterogeneous population where some of the professional groups are large and others are small. Further, the study design required that it would be possible to compare the results from this survey with those from the previous full population baseline survey. The population of HCPs in this postimplementation study was smaller than in the baseline study [1]: approximately 500 fewer individuals. The main reason for this is that the list of institutional email addresses that we received from Region Skåne had been revised and updated in the meantime; the list no longer included summer employees, for instance.

### Survey Administration

We used the Web survey tool Sunet Survey (Artisan Global Media). The emails were sent from Lund University, Lund, Sweden. On March 14, 2017, we sent a prenotification email to the study population, and on March 16, we sent a cover letter with a link to the online survey to the institutional email addresses of the professionals. Both the prenotification email and cover letter informed the recipients that participation was voluntary, that the computer files with the results were confidential, that respondents could terminate their participation at any time, and that tracking of individual responses was not possible. We did not offer any survey incentives. We sent 4 reminders, and the survey closed on April 22, 2017.

### Data Analysis

We present descriptive information for each fixed-choice question in the postimplementation survey and chi-square tests to examine the relationships between professionals and their attitudes to the Open Notes Service. Due to the small number of respondents, we grouped occupational therapists, physical therapists, social workers, unit managers, and those who selected “other” together for the chi-square tests. All reported *P* values were 2-sided. We considered *P*<.05 as statistically significant. However, in this survey, we offered response options such as “not relevant” and “I do not know,” and due to this, we did not conduct chi-square tests on 25 of the questions. In some cases, though, we did consider the question results for specific professions despite the lack of a chi-square result so that we would be able to compare the results from certain professions in the baseline study with those in the postimplementation study. The survey data were imported into and analyzed in IBM SPSS, version 23 (IBM Corporation). We also present the results of 2 of the independent open-ended questions on how the service influenced patient groups with different diagnoses. We will present the rest of the free-text responses in a separate paper. The study consisted of 2 surveys, and one of the aims was to compare the answers between them. Thus, we compared the results from the 28 questions that were similar in both surveys on a group level, and we present an overview with descriptive data of the comparison between the expectations before the implementation and the experiences after. However, in some cases where the response options differed in the second survey, we present these options together with the comparison.

### Ethics

We followed the guidelines on research ethics issued by the Swedish Research Council [9]. This study did not cover any sensitive information and did not require ethical approval according to the Swedish regulations on research ethics. Potential respondents were provided with information about the survey and its purpose in the prenotification email and the cover letter, including that participation was voluntary.

## Results

### Survey Respondents

The response rate to the Web survey was 27.73% (699/2521). The distribution of the various professions corresponded well with the overall percentage of employees in each profession in the region. The questionnaire was distributed to both permanent and temporary employees, which may have influenced the response rate negatively. Table 1 presents the demographics of the survey respondents. For statements that evaluated attitudes and experiences, we combined the alternative responses “somewhat agree” and “agree,” indicating that the respondent agreed at some level.

### Open Notes’ Influence on Patients

Among the respondents, 40.1% (276/689) estimated that 1% to 25% of their patients read their Open Notes; 37.7% (260/689) could not estimate the proportion because very few of their patients ever mentioned it. The pattern was similar for the question about how often patients brought up and talked about something they had read in their Open Notes: 42.4% (291/687) of the respondents answered that this situation occurred less than monthly, and 41.5% (285/687) answered that none of their patients had talked about the content of their health record (data not shown).

Table 2 presents the percentage of respondents who answered that they “somewhat agree” or “agree” with statements about how Open Notes influenced their patients. The first 7 statements are related to the potential benefits of Open Notes to patients, and the last 2 are related to the potential risks.

The HCPs who responded to the postimplementation survey generally thought that the expected benefits of Open Notes for patients were somewhat absent. Only 5.7% (37/651) answered that patients who read their Open Notes took better care of themselves, and 8.0% (52/650) answered that patients who read their Open Notes were more likely to take medication as prescribed. Approximately one-fourth (153/652, 23.5%) agreed to the statement that patients who read their Open Notes from psychiatry felt more in control of their health, and 14.2% (91/642) of the respondents experienced that patients who read their Open Notes trusted their HCPs more as professionals. At the same time, 24.6% (159/647) believed that patients who read their Open Notes found the notes more confusing than helpful, and 33.5% (217/648) thought that these patients worried more.

**Table 1:** Demographic characteristics of the respondents (n=699).

Characteristics		Responses, n (%)
**Professional affiliation (n=684)**		
	Nurse	191 (27.9)
	Assistant nurse	164 (24.0)
	Doctor	97 (14.2)
	Psychologist	63 (9.2)
	Medical secretary	35 (5.1)
	Social worker	45 (6.6)
	Occupational therapist	18 (2.6)
	Physical therapist	17 (2.5)
	Unit manager	28 (4.1)
	Other	26 (3.8)
**Sex (n=682)**		
	Female	492 (72.1)
	Male	154 (22.6)
	Do not want to define	36 (5.3)

**Table 2:** Psychiatric professionals’ views on how patient online access to the Open Notes Service in adult psychiatric care influenced their patients, in answer to the question stub “Generally, my patients who read their Open Notes from psychiatry online:”

Survey item	Responses, n (%)^a^
Understand their health and medical conditions better (n=657)	100 (15.2)
Remember the plan for their care better (n=649)	132 (20.3)
Take better care of themselves (n=651)	37 (5.7)
Are more likely to take medications as prescribed (n=650)	52 (8.0)
Feel more in control of their health care (n=652)	153 (23.5)
Are better prepared for appointments (n=246)	107 (16.6)
Trust me more as their care personnel (n=642)	91 (14.2)
Worry more (n=648)	217 (33.5)
Find the notes to be more confusing than helpful (n=647)	159 (24.6)

^a^Respondents who indicated “somewhat agree” or “agree” on a 4-point scale, with response options “disagree,” “somewhat disagree,” “somewhat agree,” and “agree.” There was also the option “I do not know.” We did not conduct a chi-square test on these questions due to the response options.

Of the 699 HCPs, 212 (30.3%) responded with free text to the question “For which patient groups or diagnoses in adult psychiatry may Open Notes be an asset?” There were many different responses to this open-ended question, and the most common ones were everyone (63/212, 29.7%), I do not know (26/212, 12.3%), and no one (21/212, 9.9%). Some respondents answered with examples of specific patient groups or diagnoses, and the most common answers were patients in general psychiatry (n=11), patients who need memory support (n=10), patients who understand that they are sick (n=9), and patients with depression (n=7).

Of the 699 HCPs, 276 (39.5%) responded with free text to the question “For which patient groups or diagnoses in adult psychiatry may Open Notes be particularly problematic?” The most common answers to this question were patients with a personality disorder (88/276, 31.9%), patients with psychosis (82/276, 31.1%), and patients with paranoia (47/276, 17.0%). Some respondents answered everyone (n=14), I do not know (n=13), or no one (n=11). Thus, the pattern of answers to the question about Open Notes being problematic differed from that about Open Notes being an asset.

### Changes in Practice

Table 3 shows how the respondents experienced that Open Notes influenced their practice. Generally, the results indicate that there was little impact on practice. Only 14.5% (86/594) answered that appointments took longer when patients had read their Open Notes, and 18.0% (106/588) answered that they spent significantly more time addressing patient questions outside of appointments when patients had read their notes online. Few HCPs (34/609, 5.6%) answered that Open Notes had replaced other types of communication such as letters or phone calls.

Almost one-fourth (180/671, 26.8%) of the HCPs encouraged patients to read their Open Notes, and 15.6% (105/671) of the respondents stated that they took the initiative to talk with a patient about something they were able to read in their Open Notes. Some HCPs (67/670, 10.0%) also actively used Open Notes in treatment.

The chi-square tests showed that experiences differed among the various groups of professionals. Medical secretaries (7/22, 32%) and doctors (19/86, 22%) stated more often than psychologists (6/55, 11%) and nurses (15/167, 9.0%) that appointments took significantly longer when the patient had read his or her Open Notes. The pattern was the same for the statement “I spend significantly more time addressing patient questions outside of appointments when patients have read their Open Notes.” Approximately one-third of the doctors (28/83, 34%) and 23% (5/22) of the medical secretaries answered yes to this question, compared with 15.4% (26/169) of the nurses and 14% (7/52) of the psychologists. Medical secretaries (5/26, 19%) answered more often than doctors (5/86, 6%) and nurses (4/168, 2.4%) that Open Notes had replaced other types of communication such as letters or phone calls.

### Changes in Clinical Documentation, Work Conditions, and Care Delivery

Table 4 shows the HCPs’ views on how the Open Notes Service influenced clinical documentation. Table 5 shows HCPs’ statements about how work conditions and care delivery in adult psychiatric care were influenced by this service, which is aimed at the patients. Approximately 20% of the respondents stated that they were less candid in their documentation (147/667, 22.0%) and spent more time editing notes (117/662, 17.7%) after the implementation of Open Notes. The Swedish Public Access to Information and Secrecy Act states that parts of the content in the health record may be withheld from a patient if it has been determined that the patient’s condition would deteriorate seriously if he or she were allowed to read the content. Content can also be withheld if another person (eg, a relative) is mentioned in the health record, and if that person could be endangered if the patient is allowed to read this entry. Thus, the health care provider has an obligation to carry out what is referred to here as a confidentiality check before the patient is allowed access to the information in his or her health record. Historically, this check was performed when a patient ordered a paper copy of the health record. However, the introduction of Open Notes has changed this procedure, and now all HCPs who enter documentation in health records need to carry out this confidentiality check each time they make an entry, since the patient has immediate access to the content. One-third (231/664, 34.8%) of the HCPs reported that they did a confidentiality check. At the same time, few HCPs (43/667, 6.4%) used the Specific Information template in Open Notes, which is where an HCP can enter content that is hidden from the patient online.

Due to the answer options, it was not possible to conduct a statistical analysis to examine the relationships between professionals and attitudes on the questions about clinical documentation. However, the psychologists (39/63, 62%) and doctors (36/94, 38%) responded that they were less candid in their documentation after the implementation of Open Notes. Most of the psychologists (39/62, 63%) and doctors (51/94, 54%) answered that they conducted a confidentiality check when writing in the health records, compared with 30.9% of the nurses (58/188) and 20.6% of the assistant nurses (33/160).

Table 5 presents the HCPs’ views on how Open Notes influenced work conditions and care delivery. Few HCPs (95/642, 14.8%) agreed that it changed the relationship between their profession and the patient. However, 22.8% (146/639) stated that it increased the risk for threats and violence. Only 13.2% of the HCPs (80/606) believed that patient satisfaction improved after the implementation of Open Notes, and 23.3% (141/606) believed that patient care was safer. Approximately one-third (226/668, 33.9%) of the HCPs agreed with the statement “In the future, patients should be able to write a divergent opinion that is stored in connection to the HCP’s note in the health record.” Nearly 40% of the HCPs (239/650, 36.8%) answered that Open Notes in psychiatry was a good idea.

Medical secretaries (8/26, 31%) believed that Open Notes changed the relationship between them and the patients to a larger degree than doctors (18/92, 20%) and nurses (20/181, 11.1%). Doctors (22/95, 23%) and psychologists (17/61, 28%) were less likely than assistant nurses (58/157, 37%) and nurses (74/185, 40%) to agree with the statement “Patient online access to their Open Notes in adult psychiatry is generally a good idea.”

**Table 3:** Psychiatric professionals’ views on how patient online access to the Open Notes Service in adult psychiatric care influenced their practice and results of the chi-square test for some of these items.

Survey item		Responses, n (%)	*P* value
Appointments take significantly longer time when the patient has read their Open Notes. (n=594)^a^		86 (14.5)	.005
I spend significantly more time addressing patient questions outside of appointments when patients have read their Open Notes. (n=588)^a^		106 (18.0)	.001
Has Open Notes replaced other communication such as letters or phone calls? (n=609)^a^		34 (5.6)	.002
Has the ombudsman function in Open Notes replaced other communication with relatives? (n=589)^a^		43 (7.3)	.02
Have you taken the initiative to talk with any of your patients about something they have been able to read in their Open Notes? (n=671)^b^		105 (15.6)	N/A^c^
Have you encouraged the patient to read their Open Notes? (n=671)^b^		180 (26.8)	N/A
Have you used Open Notes actively in treatment? (n=670)^b^		67 (10.0)	N/A
Psychiatric patients who read their Open Notes are more involved in their care. (n=635)^d^		125 (19.7)	.31
How often do you meet patients who have read their health record on paper? (n=651)^e^		24 (3.7)	N/A
**How many of your patients who have read their Open Notes have been offended? (n=627)^f^**			
	No patients	340 (54.2)	N/A
	1-3 patients	247 (39.4)	N/A
	4-10 patients	33 (5.3)	N/A
	11 or more patients	7 (1.1)	N/A
**How often do patients contact you or your department with questions about the contents of their Open Notes? (n=675)^f^**			
	I do not know any patients who have contacted me or my unit about this	316 (46.8)	N/A
	Less than once a month	322 (47.7)	N/A
	1-3 times a month	31 (4.6)	N/A
	1-6 times a week	4 (0.6)	N/A
	Daily	2 (0.3)	N/A
**How often are patients opposed to the contents of their Open Notes? (n=674)^f^**			
	I do not know any patients who have contacted me or my unit about this	325 (48.2)	N/A
	Less than once a month	286 (42.4)	N/A
	1-3 times a month	57 (8.5)	N/A
	1-6 times a week	4 (0.6)	N/A
	Daily	2 (0.3)	N/A
**How often do patients contact you or your department and demand that the contents of their Open Notes should be changed? (n=667)^f^**			
	I do not know any patients who have contacted me or my unit about this	398 (59.7)	N/A
	Less than once a month	244 (36.6)	N/A
	1-3 times a month	22 (3.3)	N/A
	1-6 times a week	2 (0.3)	N/A
	Daily	1 (0.1)	N/A
**How often do patients contact you or your department because they found significant errors in their EHR^g^? (n=675)^f^**			
	I do not know any patients who have contacted me or my unit about this	444 (65.8)	N/A
	Less than once a month	210 (31.1)	N/A
	1-3 times a month	18 (2.7)	N/A
	1-6 times a week	3 (0.4)	N/A
	Daily	0	N/A

^a^Respondents indicating “yes.” It was also possible to answer “no.”

^b^Respondents indicating “yes.” It was also possible to answer “no” or “not relevant.” We did not conduct a chi-square test on these questions due to the answer options.

^c^N/A: not applicable.

^d^Respondents indicating “somewhat agree” or “agree” on a 4-point scale, with response options “disagree,” “somewhat disagree,” “somewhat agree,” and “agree.”

^e^Respondents indicating that they to “a large extent” or “a very large extent” agree. It was also possible to choose the options to “a little extent,” “not at all,” or “not relevant.” We did not conduct a chi-square test on this question due to the answer options.

^f^We did not conduct a chi-square test on this question due to the answer options.

^g^EHR: electronic health record.

**Table 4:** Psychiatric professionals’ views on how patient online access to the Open Notes Service in adult psychiatric care influenced clinical documentation.

Survey item	Responses, n (%)
I am less candid in my documentation after the implementation of Open Notes. (n=667)^a^	147 (22.0)
I spend significantly more time writing or dictating or editing notes after the implementation of Open Notes. (n=662)^a^	117 (17.7)
When I write in Open Notes, I am aware that I have to do a confidentiality check because the patient can immediately read what I have written. (n=664)^a^	231 (34.8)
Do you use the Specific Information template in the health record to write information that is hidden from the patient in their Open Notes? (n=667)^a^	43 (6.4)

^a^Respondents indicating “yes.” It was also possible to answer “no” and “not relevant.” We did not conduct a chi-square test on these questions due to the answer options.

### Open Notes in the Future

Considering the continuous development and changes in the technical prerequisites of the Open Notes Service in Sweden, we found it of interest to ask the HCPs about their views on the future development of Open Notes. Approximately one-third (245/668, 36.7%) disagreed with the statement “In the future, patients should be able to write a divergent opinion that is stored in connection to the HCP’s note in the health record.” Almost as many, 33.9% (226/668), of the respondents agreed with the statement, and 29.5% (197/668) reported that they did not have an opinion. Doctors (57/96, 59%) disagreed with the statement to a higher degree than the other professional groups. Furthermore, 79 of 699 HCPs (11.3%) responded with free text to the question “Do you have any suggestions to improve Open Notes, to make the service more useful to patients and health care providers?” Here are some examples of the suggestions: HCPs should be able to sign a note in the health record with an identification number instead of name in order to feel safer; HCPs should be notified that a patient has read his or her Open Notes; HCPs should be able to decide when a note should be visible online. The HCPs also stated that there should be more education and information about the service for both the employees and the patients, and that it should be possible for patients and their relatives to write their views of problems in the health record. However, there were also respondents who suggested that the service should be shut down, and that the health record had primarily been a tool for the HCPs in their work in the past and that it should have remained that way.

**Table 5:** Psychiatric professionals’ views on how patient online access to the Open Notes Service in adult psychiatric care influenced work conditions, and the results of the chi-square test for some of these items.

Survey item	Responses, n (%)	*P* value
Medical care is delivered more efficiently after the implementation of Open Notes. (n=616)^a^	74 (10.6)	.04
Patient satisfaction has improved after the implementation of Open Notes. (n=606)^a^	80 (13.2)	.02
Oral reporting between staff has increased since the implementation of OpenNotes. (n=641)^a^	72 (11.2)	.009
Patient care is safer after the implementation of Open Notes. (n=606)^a^	141 (23.3)	.86
Patient online access to their Open Notes contributes to health care on equal terms for all patients. (n=640)^b^	119 (18.6)	.71
Patient online access to their Open Notes in adult psychiatry influences the relationship between the different professions working there. (n=638)^b^	63 (9.9)	.48
Patient online access to their Open Notes in adult psychiatry influences the relationship between the patient and your profession. (n=642)^b^	95 (14.8)	<.001
Patient online access to their Open Notes in adult psychiatry influences the risk for me to be subjected to threat and violence. (n=639)^c^	146 (22.8)	N/A^d^
Patient online access to their Open Notes in adult psychiatry influences the risk for me to be reported to the Patients Advisory Committee. (n=635)^c^	145 (22.8)	N/A
Patient online access to their Open Notes in adult psychiatry influences the risk for me to be reported to the Health and Social Care Inspectorate. (n=631)^c^	103 (16.3)	N/A
In the future, patients should be able to write a divergent opinion that is stored in connection to the HCP’s^e^ note in the health record. (n=668)^f^	226 (33.9)	N/A
Patient online access to their Open Notes in adult psychiatry is generally a good idea. (n=650)^g^	239 (36.8)	.005

^a^Respondents indicating “yes”. It was also possible to answer “no”.

^b^Respondents indicating that agree to “a large extent” or “a very large extent.” It was also possible to choose the options to “a little extent” or “not at all.”

^c^Respondents indicating that “the risk will increase.” It was also possible to answer “the risk will not change,” “the risk will decrease,” and “not relevant.” We did not conduct a chi-square test on these questions due to the response options.

^d^N/A: not applicable.

^e^HCP: health care professional.

^f^Respondents who answered “somewhat agree” or “agree” on a 4-point scale, with response options “disagree,” “somewhat disagree,” “somewhat agree,” and “agree.” It was also possible to answer “no opinion.”

^g^Respondents who answered “somewhat agree” or “agree” on a 4-point scale, with response options “disagree,” “somewhat disagree,” “somewhat agree,” and “agree.”

### Comparison Between Results From the Baseline Survey and the Postimplementation Survey

Table 6 presents an overview of the results from the 2 surveys that are comparable with each other. Part 1 of this report shows detailed information about the responses from the baseline survey [1], and this paper reports detailed information about the responses from the postimplementation survey above.

The general tendency in the comparisons is that approximately half as many respondents in some way agree with the statements in the postimplementation survey as in the baseline survey. However, there are some exceptions. The first 4 statements in the changes in practice section show that many respondents in the baseline survey expected that patients would be more active in different ways when they had read their Open Notes. The results from the second survey, however, show that very few patients contacted the HCPs with questions, requested changes to the content, found significant errors, or opposed what was written in the notes. Another comparison that does not follow the general tendency is the statement about patients being offended. The results from the 2 surveys are almost the same, but it is important to note that it was unusual that the respondents met patients who were offended. The more detailed results in Table 3 show that most of these HCPs stated that they had met 1 to 3 patients who were offended. Another result that is almost the same in both surveys is that Open Notes contributed to health care on equal terms for all patients: respondents in both surveys agreed with this statement to the same extent. Yet another difference can be found in the last statement in Table 6 (Open Notes in adult psychiatry is generally a good idea), where the percentage was higher in the postimplementation survey than in the baseline survey.

**Table 6:** Comparison between results from the baseline survey and the postimplementation survey.

Survey item		Baseline^a^, %	Postimplementation, %
**Influence on patients**			
	Patients will/did understand their health and medical conditions better.	30.1^b^	15.2^b^
	Patients will/did remember the plan for their care better.	48.2^b^	20.3^b^
	Patients will/did take better care of themselves.	11.2^b^	5.7^b^
	Patients will be/are more likely to take medications as prescribed.	18.5^b^	8.0^b^
	Patients will/did feel more in control of their health care.	44.4^b^	23.5^b^
	Patients will be/are better prepared for visits.	31.1^b^	16.6^b^
	Patients will/did trust me more as their caregiver.	27.4^b^	14.2^b^
	Patients will/did worry more.	58.1^b^	33.5^b^
	Patients will/did find the notes to be more confusing than helpful.	52.7^b^	24.6^b^
**Changes in practice**			
	Patients will/did contact me or my practice with questions about their notes.	68.7^b^	5.5^c^
	Patients will/did find significant errors in the notes.	41.9^b^	3.1^c^
	Patients will/did oppose with what is written in their notes.	63.2^b^	9.4^c^
	Patients will/did request changes to the content of notes.	52.4^b^	3.7^c^
	Patients will be/are offended.	44.5^d^	45.8^e^
	Visits will take/take longer.	35.1^d^	14.5^f^
	I will spend/spend time addressing patient questions outside of visits.	40.6^d^	18.0^f^
**Changes in clinical documentation, work condition, and care delivery**			
	I will be/am less candid in my documentation.	40.5^d^	22.0^f^
	I will spend/spend more time writing/dictating/editing notes.	41.5^d^	17.7^f^
	Medical care will be/is delivered more efficiently.	21.1^f^	10.6^f^
	Patient satisfaction will/has improve(d).	29.5^f^	13.2^f^
	Patient care will be/is safer.	36.3^f^	23.3^f^
	Open Notes will contribute/contribute to health care on equal terms for all patients.	17.3^g^	18.6^g^
	Open Notes will influence the relationship between professions.	20.6^g^	9.9^g^
	Open Notes will influence the relationship between the patient and your profession.	35.6^g^	14.8^g^
	Open Notes will influence the risk for me to be subjected to threat and violence.	45.6^h^	22.8^h^
	Open Notes will influence the risk for me to be reported to the Patients Advisory Committee.	42.2^h^	22.8^h^
	Open Notes will influence the risk for me to be reported to the Health and Social Care Inspection.	32.2^h^	16.3^h^
	Open Notes in adult psychiatry is generally a good idea.	27.7^b^	36.8^h^

^a^Published previously in part 1 [1].

^b^Percentage of respondents who indicated “somewhat agree” or “agree.”

^c^Percentage of respondents who indicated “1-3 times a month,” “1-6 times a week” or “daily.”

^d^Percentage of respondents who indicated that they were “moderately concerned,” “very concerned,” or “so concerned that, I do not want Open Notes in psychiatric care at all.”

^e^Percentage of respondents who indicated “1-3 patients,” “4-10 patients,” or “11 or more patients.”

^f^Percentage of respondents who indicated “yes.”

^g^Percentage of respondents who indicated that they to “a large extent” or “a very large extent” agree.

^h^Percentage of respondents who indicated that “the risk will increase.”

## Discussion

### Principal Findings

To our knowledge, the previously published paper, with resulted from the preimplementation baseline survey [1], was the first of its kind to examine how HCPs working in adult psychiatric care in a universal health care setting anticipated how Open Notes would influence their work and their patients. Accordingly, our postimplementation survey is the first follow-up survey of its kind to examine how HCPs working in this setting experienced the influence of Open Notes on their patients, their work, and care delivery. The 2 surveys make it possible to compare and discuss the differences between the expectations and the experiences of the implementation of the Open Notes Service.

One of the main arguments for Open Notes is that the service will increase patient participation and, hence, patient empowerment. The rhetoric and expectations from politicians and key actors are, for example, that patients who read their notes will take better care of themselves, that they will feel more in control of their care, and that they will be better prepared for appointments [2]. Thus, in the 2 surveys, we asked the HCPs in adult psychiatric care about their expectations and experiences of both the benefits and risks for their patients. Comparison of the results (Table 6) shows that both hopes about benefits and worries about risks for patients were higher before the Open Notes implementation than after. Thus, Table 6 shows that the proportion of respondents who agreed with the statements about how Open Notes would influence patients in the postimplementation survey is approximately half as large as in the baseline survey [1]. There were other differences between the 2 surveys: doctors, psychologists, and medical secretaries were in many cases more negative toward the service than were nurses and assistant nurses in the baseline survey. In the postimplementation survey, though, there were fewer such differences in opinions between the various professional groups. The respondents in the postimplementation survey also stated that, in general, there had been little actual impact on their practice in terms of longer appointments or more questions from patients about the contents of their health records.

One explanation for these results may be that many patients in adult psychiatry in Region Skåne did not read their Open Notes. Almost one-third of the respondents in the baseline survey [1] thought that 50% or more of their patients would read them online. However, the results of the postimplementation survey show that very few HCPs met patients who mentioned they had read their Open Notes. One reason may be that little or no information had been given to patients in adult psychiatric care in Region Skåne about the service. This is different from other countries: patients in Sweden, for example, do not receive an email from their HCP notifying them that there is a new online note to read. Thus, Swedish patients in Region Skåne may not be aware that they are able to read notes from a psychiatry appointment online. Another explanation could be that some patients had read their notes but neglected to mention it to the HCPs.

The introduction of Open Notes in Sweden is widespread. We wondered whether the Region Skåne HCPs in our postimplementation survey had any thoughts about how the service could influence different groups of patients in psychiatric care. We asked them whether they thought that Open Notes benefited any specific group or might be particularly problematic for others. The most common free-text answer about which patients would benefit was everyone, followed by I do not know and no one. They thus responded with general attitude statements about the service, rather than mentioning specific patient groups. This indicates that some respondents seemed to be either generally positive toward the Open Notes Service or generally negative toward it. This observation is strengthened by the fact that these free-text answers, which can be described as almost ideological statements, also appeared among the other free-text answers. However, research showed that employees who are dissatisfied are more likely to answer open-ended questions [10], and this should be taken into consideration. Still, such ideological standpoints may influence how professionals act in their daily work. However, the pattern was very different in the answers to the question about Open Notes being particularly problematic for some groups of patients. The 2 most common answers to this free-text question were “patients with a personality disorder” and “patients with psychosis;” “patients with paranoia” was the third most common answer. This result points out a delicate problem: all patients, regardless of diagnoses, have the same right to read their health records online. However, the answers from the HCPs indicate that the transparency could be more problematic for some patients than for others. These opinions could influence how HCPs who meet patients with these diagnoses act in their work. This can be connected to the results from both surveys, which indicate that Open Notes might change clinical documentation. We cannot but wonder whether the HCPs treating patients with the 3 diagnoses above were overrepresented in the group of HCPs who claimed that they became less candid in their documentation; further research is needed to clarify this question.

One of the most interesting results in the baseline survey was that approximately 60% of both doctors and psychologists were worried that they would be less candid in their documentation after the implementation of Open Notes. Findings from studies in the United States indicate the same concerns, with concerns that the increased transparency would “water down” the content in the records [11]. Mental health clinicians in the United States also claimed that they were more careful about what they wrote to protect themselves and their patients [12]. The results from the postimplementation survey indicate that there have been changes in clinical documentation: over 60% of the psychologists and nearly 40% of the doctors stated that they were less candid in their documentation after the implementation of Open Notes. This indicates that there may be a risk for watered down health records in adult psychiatry, and it is important to gain a greater understanding of when these situations occur and why there are HCPs who changed their way of writing entries in the health record after the implementation of Open Notes. Yet another perspective is that less candid information in the health records could negatively influence both the work of the HCPs and the overall aim to have more informed and active patients. It may become more difficult for HCPs to deliver safe care with high quality if information is missing in the health records, and the vision of increased patient participation can be harder to fulfill if important information is withheld from a patient because of less candid documentation.

Furthermore, patient safety is an important part of care, and new ways of delivering care may influence patient safety in different ways. An overall and important intention in health care is to increase patient safety; hence, an interesting question is whether Open Notes does that. Table 6 shows that 36.3% of the HCPs in the baseline survey thought that implementation would increase patient safety. Among the respondents who answered the postimplementation survey, though, only 23.3% stated that patient safety increased after implementation. These results, together with the rest of the results, indicate that the enhanced transparency may influence patient safety in different ways. Thus, there is a need for more knowledge about the influence of the Open Notes Service on patient safety.

Finally, it is important to note that an implementation of such a service as Open Notes can never be described as completed. There will always be new patients and new employees who will have to learn to use the service, and the prerequisites of the technology will change due to new regulations and new, more up-to-date electronic health records. Consequently, there will always be old users who will have to learn to manage new technical prerequisites, and new users who will have to learn to manage the existing ones. In addition, there will always be patients who never read their Open Notes for different reasons. Being aware of all these circumstances, we decided that the second survey would be distributed one and a half years after the implementation of Open Notes in adult psychiatric care, so that the employees would have had considerable experience of both using the service and meeting patients who had read their records online. Still, many employees stated that they never met a patient who said that he or she had done so, but one important result from the postimplementation survey is that the employees acted as if the patients had read their notes online. Thus, Open Notes seems to have changed the clinical documentation irrespective of whether the patients were active.

### Limitations

This study has several limitations. First, we had no way of knowing whether the same individuals answered the baseline survey and the postimplementation survey. It was not possible to send the survey to the same individuals because this was a full population study, and during the one and a half years between the 2 surveys, new employees started working in adult psychiatric care and others left. Thus, it is possible to compare the results from the 2 surveys on a group level, but we do not know whether and how individual employees did or did not change their opinion about Open Notes. Second, the response rate to the Web questionnaire was 27.73%. This is similar to the response rate in the baseline survey of 28.86% [1]. The explanations for the response rate may be the same as in the previous survey: some employees may have been absent during the time that it was possible to answer the survey, and the implementation of Open Notes may have been a sensitive topic that could have influenced the response rate negatively. A third limitation, unfortunately, is that there were no separate statistics for the log-ins by patients in psychiatric care. Fourth, we were not able to conduct chi-square tests on as many answers in the postimplementation survey as in the baseline survey. This was due to the answer options “not relevant” and “I do not know” for some of the questions. Thus, it was not possible to carry out statistical analyses to examine the relationships between professionals and their attitudes to the Open Notes Service to the same extent as in the first survey.

### Conclusions

Given the expansion of the use of Open Notes in psychiatry [13,14], there is a need for more knowledge about how the technology influences both patients and HCPs, since the technology, at least in Sweden, primarily was developed and deployed for nonpsychiatric settings. Consequently, some HCPs have worried that Open Notes in psychiatry would put both patients and themselves at risk [1,14]. Through the analysis and comparison of the baseline survey and the postimplementation survey, we have been able to both provide more knowledge to this important area and identify areas where even more is needed.

First, the results of this study show that most HCPs who answered the postimplementation survey did not experience that patients in adult psychiatric care were more involved in their care after the implementation of Open Notes. Thus, it would be interesting to study the patients’ views of Open Notes and compare the results with those from this study.

Second, the postimplementation survey results also indicate that the clinical documentation changed after the implementation of Open Notes. This implies that a technical solution aimed at the patients may change documentation patterns in a way that neither increases care safety nor establishes good conditions for HCPs to deliver high-quality care. This might be particularly problematic, as doctors and psychologists were the 2 main groups that claimed that they became less candid in their documentation after the implementation of Open Notes. This also indicates a need for more knowledge about the actual influence of Open Notes in terms of changes in the content of health records.

Third, the results indicate that it may be important to prepare HCPs before an implementation of Open Notes, especially in medical areas where the service can be considered sensitive. This is an actual issue at the moment in Region Skåne, since the plan is that patients in forensic psychiatry, parents of patients in child and youth psychiatry who are younger than 13 years, and adolescents who are older than 16 years will be able to read health records from psychiatry online from the fall of 2018.

Fourth, further research is needed to investigate the influence of Open Notes in psychiatric settings over time, especially regarding changes in clinical documentation. There is a need for studies that focus on how Open Notes influence patient safety in both psychiatric and nonpsychiatric settings. Research is also needed on how Swedish patients, especially in psychiatric care, use their Open Notes.

## Abbreviations

EPSA: eHealth Services’ Impact on the Working Environment of Health Professionals
EHR: electronic health record
HCP: health care professional

## References

[1] Petersson L, Erlingsdóttir G (2018). Open Notes in Swedish psychiatric care (part 1): survey among psychiatric care professionals. JMIR Ment Health.

[2] Erlingsdóttir G, Lindholm C (2015). When patient empowerment encounters professional autonomy: the conflict and negotiation process of inscribing an eHealth service. Scand J Public Adm.

[3] Grünloh C, Cajander Å, Myreteg G (2016). “The record is our work tool!”-physicians' framing of a patient portal in Sweden. J Med Internet Res.

[4] Grünloh C, Myreteg G, Cajander Å, Rexhepi H (2018). “Why do they need to check me?” Patient participation through eHealth and the doctor-patient relationship: qualitative study. J Med Internet Res.

[5] Delbanco T, Walker J, Bell SK, Darer JD, Elmore JG, Farag N, Feldman HJ, Mejilla R, Ngo L, Ralston JD, Ross SE, Trivedi N, Vodicka E, Leveille SG (2012). Inviting patients to read their doctors' notes: a quasi-experimental study and a look ahead. Ann Intern Med.

[6] Walker J, Leveille SG, Ngo L, Vodicka E, Darer JD, Dhanireddy S, Elmore JG, Feldman HJ, Lichtenfeld MJ, Oster N, Ralston JD, Ross SE, Delbanco T (2011). Inviting patients to read their doctors' notes: patients and doctors look ahead: patient and physician surveys. Ann Intern Med.

[7] Delbanco T, Walker J, Darer JD, Elmore JG, Feldman HJ, Leveille SG, Ralston JD, Ross SE, Vodicka E, Weber VD (2010). Open notes: doctors and patients signing on. Ann Intern Med.

[8] Walker J, Delbanco T (2013). Interval examination: moving toward open notes. J Gen Intern Med.

[9] Swedish Research Council (2017). Good Research Practice.

[10] Poncheri R, Lindberg J, Foster TL, Surface E (2008). A comment on employee surveys. Negativity bias in open-ended responses. Organ Res Methods.

[11] Walker J, Darer JD, Elmore JG, Delbanco T (2014). The road toward fully transparent medical records. N Engl J Med.

[12] Denneson LM, Cromer R, Williams HB, Pisciotta M, Dobscha SK (2017). A qualitative analysis of how online access to mental health notes is changing clinician perceptions of power and the therapeutic relationship. J Med Internet Res.

[13] Essén A, Scandurra I, Gerrits R, Humphrey G, Johansen M, Kiergegaard P, Koskinen J, Liaw S, Odeh S, Ross P, Ancker J (2018). Patient access to electronic health records: differences across ten countries. Health Policy Technol.

[14] Dobscha SK, Denneson LM, Jacobson LE, Williams HB, Cromer R, Woods S (2016). VA mental health clinician experiences and attitudes toward OpenNotes. Gen Hosp Psychiatry.

